# Comparative and Evolutionary Analysis of the Bacterial Homologous Recombination Systems 

**DOI:** 10.1371/journal.pgen.0010015

**Published:** 2005-08-26

**Authors:** Eduardo P. C Rocha, Emmanuel Cornet, Bénédicte Michel

**Affiliations:** 1 Unité Génétique des Génomes Bactériens, Institut Pasteur, Paris, France; 2 Atelier de Bioinformatique, Université Pierre et Marie Curie, Paris, France; 3 Laboratoire de Génétique Microbienne, Institut National de la Recherche Agronomique, Jouy en Josas, France; MRC Cancer Cell Unit, United Kingdom

## Abstract

Homologous recombination is a housekeeping process involved in the maintenance of chromosome integrity and generation of genetic variability. Although detailed biochemical studies have described the mechanism of action of its components in model organisms, there is no recent extensive assessment of this knowledge, using comparative genomics and taking advantage of available experimental data on recombination. Using comparative genomics, we assessed the diversity of recombination processes among bacteria, and simulations suggest that we missed very few homologs. The work included the identification of orthologs and the analysis of their evolutionary history and genomic context. Some genes, for proteins such as RecA, the resolvases, and RecR, were found to be nearly ubiquitous, suggesting that the large majority of bacterial genomes are capable of homologous recombination. Yet many genomes show incomplete sets of presynaptic systems, with RecFOR being more frequent than RecBCD/AddAB. There is a significant pattern of co-occurrence between these systems and antirecombinant proteins such as the ones of mismatch repair and SbcB, but no significant association with nonhomologous end joining, which seems rare in bacteria. Surprisingly, a large number of genomes in which homologous recombination has been reported lack many of the enzymes involved in the presynaptic systems. The lack of obvious correlation between the presence of characterized presynaptic genes and experimental data on the frequency of recombination suggests the existence of still-unknown presynaptic mechanisms in bacteria. It also indicates that, at the moment, the assessment of the intrinsic stability or recombination isolation of bacteria in most cases cannot be inferred from the identification of known recombination proteins in the genomes.

## Synopsis

Genomes evolve mostly by modifications involving large pieces of genetic material (DNA). Exchanges of chromosome pieces between different organisms as well as intragenomic movements of DNA regions are the result of a process named homologous recombination. The central actor of this process, the RecA protein, is amazingly conserved from bacteria to human. In addition to its role in the generation of genetic variability, homologous recombination is also the guardian of genome integrity, as it acts to repair DNA damage. RecA-catalyzed DNA exchange (synapse) is facilitated by the action of presynaptic enzymes and completed by postsynaptic enzymes (resolvases). In addition, some enzymes counteract RecA. Here, the researchers assess the diversity of recombination proteins among 117 different bacterial species. They find that resolvases are nearly as ubiquitous and as well conserved at the sequence level as RecA. This suggests that the large majority of bacterial genomes are capable of homologous recombination. Presynaptic systems are less ubiquitous, and there is no obvious correlation between their presence and experimental data on the frequency of recombination. However, there is a significant pattern of co-occurrence between these systems and antirecombinant proteins.

## Introduction

Homologous recombination was originally described as being the result of the sexual process—in bacteria as in eukaryotes—and was later identified as a major DNA repair process. Both genetic and biochemical studies revealed the crucial role of homologous recombination in all organisms for the repair of a variety of DNA damage of exogenous and endogenous origin [1,2]. Indeed, in all organisms in which it has been tested, inactivation of RecA causes a dramatic increase of sensitivity to all DNA-damaging agents used in laboratories. In addition to its housekeeping role in repair, recombination is fundamental for the genetic diversification of bacterial genomes. First, in bacteria it allows the integration of homologous alien DNA, arising from transformation or conjugation [3,4]. Second, by allowing allelic recombination between closely related strains [5], it assorts adaptive mutations and purges deleterious mutations hitchhiking with them [6]. Third, recombination between homologous segments in the genomes leads to chromosomal instability [7,8], and among bacteria, the rate of chromosome rearrangements correlates with the number of repeated sequences in the genomes [9]. Fourth, intrachromosomal homologous recombination between large repeated regions is often adaptive, allowing the generation of genotypic diversity, e.g., in pathogens [10–12].

The general outline of homologous recombination is common to all organisms studied to date. It involves a central step of strand-invasion and strand-exchange catalyzed by RecA or a RecA homolog. RecA is ubiquitous and highly conserved in sequence. Strand exchange is preceded by the action of enzymes called presynaptic enzymes. These enzymes act on DNA to render it accessible to RecA and thus allow the formation of a RecA filament, which is single-stranded DNA (ssDNA) coated with RecA molecules. The steps that follow strand exchange and result in the formation of a viable recombinant molecule are termed postsynaptic and are mainly the resolution of the recombination intermediate made by RecA. The entire process and the enzymes involved have been originally defined and extensively characterized in *Escherichia coli,* which has become a paradigm for homologous recombination [1,13,14]. For this reason, the *E. coli* genes were used in this work to search for homologs in other bacteria. Genes of *Bacillus subtilis,* the second model bacteria, were used for enzymes absent from *E. coli*.

**Figure 1:**
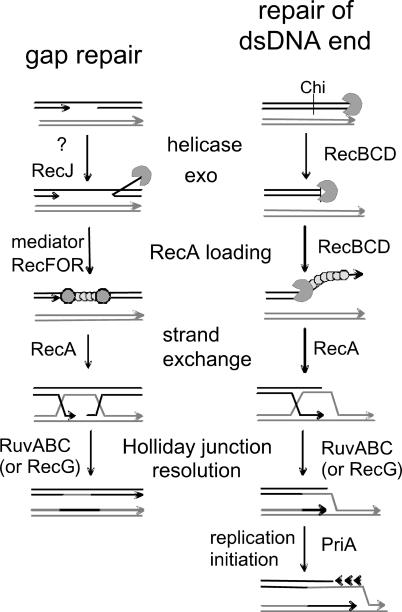
Models for the Mode of Action of the Main Homologous Recombination Proteins in *E. coli* at ssDNA Gaps or dsDNA Ends Enzymes of known biochemical activities are shown. The presynaptic steps result in the formation of a RecA filament. At gaps, this step requires RecJ, RecF, RecO, and RecR: the 5′ ssDNA exonuclease RecJ enlarges the ssDNA region (possibly with the help of various helicases, as no specific helicase is required for gap repair); RecF, RecO, and RecR promote RecA binding to SSB-coated DNA. At dsDNA ends, RecBCD (AddAB in *B. subtilis*) degrades DNA until it encounters a χ site; its helicase-nuclease activity is then modified to produce a 3′-ended ssDNA, to which it loads RecA. The synaptic step (homology search and strand exchange) is always performed by RecA and results in the formation of a Holliday junction (X structure). The postsynaptic steps are the migration and the resolution of Holliday junctions. Migration can be performed by RuvAB or by RecG, and resolution is made by RuvC (RecU in *B. subtlis;* RuvC forms a complex with RuvAB in *E. coli*). In addition, RecBCD-mediated recombination is always coupled with PriA-dependent replication restart. Antirecombinases are not shown: UvrD and MutLS prevent by different means the strand exchange reaction. In *recBC* mutants, the presynaptic steps of dsDNA end repair can be catalyzed by the helicase RecQ and the gap repair proteins RecJ and RecFOR, a reaction that is prevented by SbcB (and SbcCD) nucleases.

The initiation of homologous recombination in *E. coli* may follow the RecBCD or the RecFOR pathway (Figure 1). Both pathways work to provide a ssDNA molecule coated with RecA to allow the invasion of a homologous molecule [13,15]. RecBCD promotes the repair of double-stranded DNA (dsDNA) breaks, whereas RecFOR is involved in the repair of ssDNA gaps. In the RecBCD pathway, all the required functions—helicase, nuclease, and RecA loading—are assembled in a single holoenzyme [16]. RecBCD binds to dsDNA ends, unwinds, and degrades DNA until it encounters a χ site. The activity of RecBCD is modified at χ, where it starts producing ssDNA and loading RecA [17]. RecF, RecO, and RecR bind gapped ssDNA and displace the SSB proteins to allow RecA coating. There is evidence for interactions between RecR and either RecF or RecO, but not for the existence of a tricomponent complex [18,19]. The RecJ ssDNA exonuclease acts in concert with RecFOR to enlarge the ssDNA region when needed. Strand exchange is catalyzed by RecA [20], a multifunctional protein also involved in the regulation of the SOS response and in the activity of polymerases that facilitate replication across DNA lesions [21]. In *E. coli,* the joint molecules formed by RecA are resolved by either the RuvABC complex or, in an unknown way, by the action of the RecG helicase. The RuvAB and RuvC proteins catalyze the branch migration and the resolution of Holliday junction recombination intermediates, respectively. These three proteins are thought to interact in a resolvasome complex, in which a RuvABC-junction complex tracks along DNA, with RuvC able to scan for cleavable sequences as the DNA passes through (Figure 1). Finally, replication is directly linked to the recombination process during double-strand break repair, as a viable recombinant is only obtained if the recombination intermediate is used to initiate replication, via the action of the PriA protein [22,23]. Conversely, recombination proteins participate in replication progression as, for example, RecFOR and RecA are required for the resumption of a normal replication rate after treatment with a DNA-damaging agent, and RecBC is required for the viability of several replication mutants [2].

Evidence is accumulating that other bacteria use different proteins for some recombination steps. For example, in firmicutes, RecBCD is replaced by the analogous complex AddAB (named RexAB in streptococci and lactococci) [24,25], and there is evidence indicating that a functional χ site is present in these genomes, albeit variable in size and composition [26]. In these genomes, RecU also replaces RuvC [27]. The frequency of homologous recombination is diminished by the action of other proteins. The general mismatch repair system (MutS1LH in *E. coli*) antagonizes homologous recombination between nonidentical DNA sequences by blocking the RecA-mediated strand exchange process if mismatches are present [28]. Hence, the mismatch repair system prevents recombination between homeologous sequences and has an important role in defining bacterial species barriers [29]. The helicase II, UvrD, also acts as an antirecombinant, possibly by unwinding the paired DNA recombinant intermediates [30], or by displacing RecA from ssDNA [31]. On the other hand, UvrD can stimulate RecA-driven branch migration and can participate in the RecFOR pathway [32]. Finally, in *recBC* mutant cells, RecFOR can initiate recombination from DNA double-strand ends that have a single-strand extension, but only when SbcB, a ssDNA-specific 3′ → 5′ exonuclease, is inactivated. When present, this nuclease prevents RecFOR action by removing the 3′ extremity on which RecA could be loaded; in addition, the growth of *recBC sbcB* mutants requires the inactivation of the SbcCD proteins for unknown reasons [1,33]. Antirecombinant proteins must be taken into account when assessing the potential recombination machinery of bacteria, as it can be evaluated from genome sequences.

An extended assessment of the proteins involved in DNA repair followed the publication of the first genome sequences [34]. This pioneering work showed that genes implicated in homologous recombination are not homogeneously distributed among bacterial species. Unfortunately, no equivalent extensive work has been done recently that focuses precisely on homologous recombination and takes advantage of the nearly 200 completely sequenced genomes. Yet different sets of recombination-related genes have been found among some bacterial groups [35–38]. We have thus tried to assess the distribution of homologous recombination genes in complete genomes, using a large set of tools involving sequence and phylogenetic analysis [34,39], as well as colocalization data. This type of analysis presupposes that recombination proteins are ancient enough to have diverged from one or a few proteins for which we know the function for at least one element in the family. Although recombination is probably a very old process, our data suggest that some genes may have been missed because they are not yet functionally characterized. A further assumption of our analysis is that sequence similarity will remain strong enough to allow finding these genes by sequence similarity. We make some simulations that suggest that few genes are likely to have been lost if sequence divergence follows the pattern (but not necessarily the rate) of RecA. The use of genomic context should also reduce this problem. Finally, this analysis also supposes that orthologs have similar functions. Although this is usually assumed, proteins with multiple functions may have gained or lost part of them during evolution. For example, the role of RecA in SOS is unused—possibly lost—in the bacteria that lack this response. After establishing the repertoire of genes, we evaluate their co-occurrence, evolutionary rate, and colocalization, taking into account their functional association in known pathways. This was then put into relation with the evolutionary history of genes and the assessment of the experimental evidence for recombination.

## Results/Discussion

### Introductory Remarks

**Figure 2:**
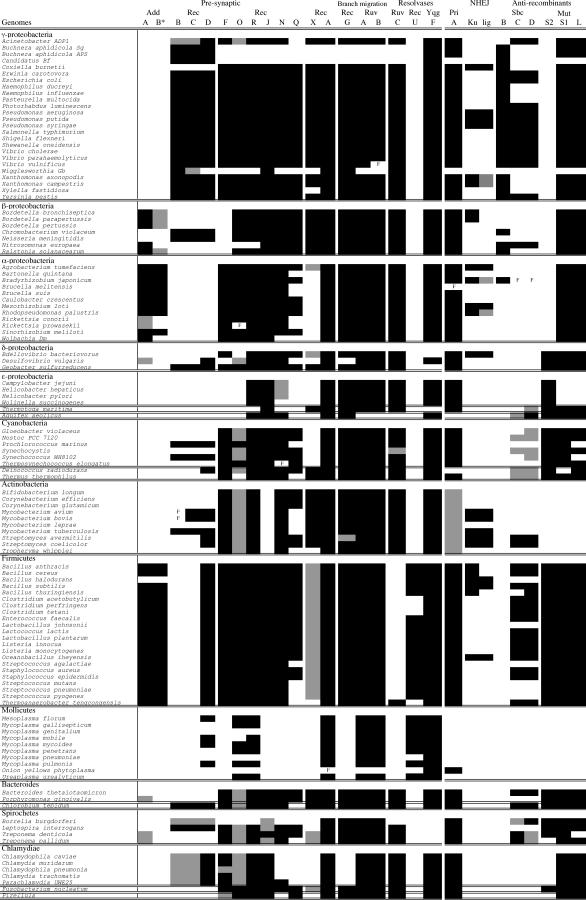
Probable Presence, Putative, and Unlikely Presence of Recombination-Associated Genes in the Studied Genomes Black indicates presence is probably, grey indicates putative presence, and white indicates presence is unlikely. F indicates that the gene is present in the genome but contains frame shifts (genes with known programmed frame shifts, introns, and inteins are indicated, as regular genes, in black).

As described in Materials and Methods, we first applied an automatic methodology to find candidate orthologs of genes implicated in homologous recombination. The analysis started from genomes for which experimental evidence was available for the function of the genes. This typically included not only *E. coli* and *B. subtilis* but also much less studied bacteria such as mollicutes (for RuvAB [40]), actinobacteria (for Ku [41]), or others. Naturally, when an ortholog was found in a phylogenetic group, it was used to search for further orthologs within the group. Second, we made a more detailed analysis by searching for InterPro domains and making FASTA searches; and by taking into account phylogenetic analyses and information on gene colocalization. Using these diverse sources of information, we were able to list candidate homologous recombination genes in 117 genomes (Figure 2). Some genes are highly conserved in sequence and nearly ubiquitous. For these genes, the methods we used are very reliable and provide uniformly consistent results. However, for some less ubiquitous, fast-evolving, or poorly characterized genes, we found sometimes either inconsistent similarity or weak hits, e.g., similarity smaller than 40%, FASTA hits with *E* ~10^−5^, matches with a nonspecific motif or with large variation in protein length. Under these conditions, and when no reliable close ortholog is available, it is hazardous to confidently predict orthology. Hence, we conservatively regard these genes as “putative” orthologs. For some proteins, e.g., RecO and RecX, the list of putatives is relatively large.

### RecA and Resolvases Are Nearly Ubiquitous Genes

**Figure 3:**
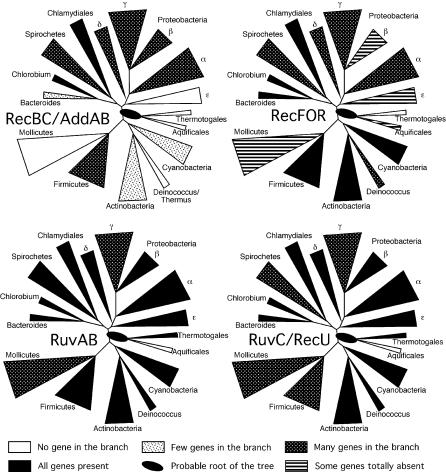
Distribution of Some Recombination Genes in a Phylogenetic Tree of Bacteria Tree adapted from [99]. The position of *Pirellula* and *Fusobacterium* are still unclear [100].

No homologous recombination gene is present in all bacterial genomes. However, many genes are widespread among all or nearly all groups and are extremely frequent within each group (Figure 2). RecA is absent only in the several genomes of *Buchnera* and *Blochmania* and presents frame shifts in Onion Yellows (OY) phytoplasma. The near ubiquity of RecA matches well with its preeminent role in homologous recombination and has been previously noticed [34,42,43]. Its absence among intracellular bacteria has also been widely documented [36,44–46]. Unsurprisingly, bacteria lacking RecA have very few other recombination proteins. Several proteins are almost as frequent as RecA. The genes coding for the RuvAB Holliday junction branch migration complex always co-occur and are absent from the genomes that lack RecA and from only two genomes where RecA is present, *Wigglesworthia* Gb and *Aquifex aeolicus* (Figures 2 and 3). Although they lack RuvAB, these two genomes contain a RecG ortholog—another Holliday junction branch migrating helicase. The gene for RecG is also very frequent, absent only from all mollicutes and all Chlamydiacea as well as from *Desulfovibrio vulgaris*.

Some proteins are believed to be functional analogs, although they apparently lack a common evolutionary history (i.e., they are not orthologs). RuvAB in *E. coli* forms a complex with the resolvase RuvC. RuvC is less ubiquitous than RuvAB, which is explained by its functional replacement by the analog RecU in firmicutes and mollicutes [27]. Our data indicate that only ten genomes lack both RuvC and RecU (this includes the genomes that lack RecA; Table 1). In these rare cases, the resolvase function may be provided by YqgF [47], which is only absent from seven genomes. However, our data suggest that RuvC/RecU and YqgF are not simple functional analogs because they co-occur in the large majority of genomes. In addition, a resolvase activity of the YqgF proteins has not yet been demonstrated either in vitro or in vivo. The function performed by resolution proteins may also be carried out by prophage-encoded proteins [48].

**Table 1:**
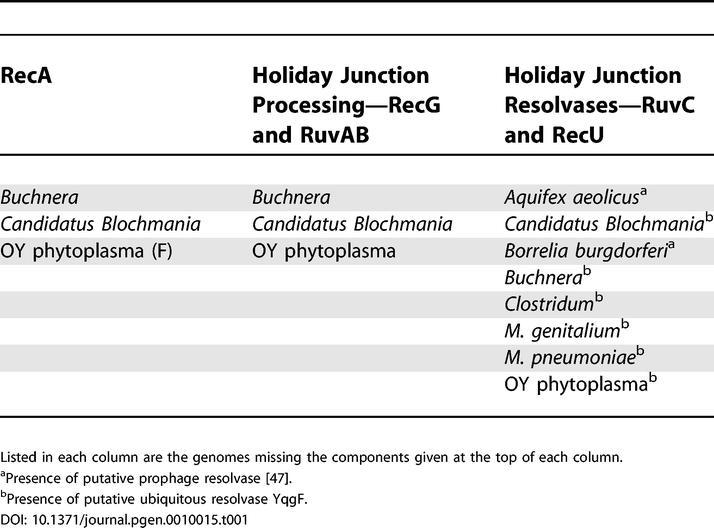
Genomes Missing Key Components of the Homologous Recombination

Listed in each column are the genomes missing the components given at the top of each column.

^a^Presence of putative prophage resolvase [47].

^b^Presence of putative ubiquitous resolvase YqgF.

PriA is nearly ubiquitous and is only absent in genomes of some intracellular endosymbionts, *Deinococcus radiodurans, A. aeolicus,* and from most genomes of mollicutes. Among actinobacteria, there is a putative ortholog of PriA that is smaller and very divergent. With the exception of *Candidatus Blochmania floridanus* (which lacks RecA), all genomes with AddAB or RecBCD (the presynaptic proteins that act at double-strand ends) have PriA. In conclusion, RecA, branch migration systems, and resolvases, and to a lesser extent the protein that couples recombination and replication PriA, are present in nearly all the bacterial genomes (Table 1).

### The RecBCD and AddAB Presynaptic Recombination Proteins

RecBCD provides another example of complementary distribution of similar but nonorthologous systems. The AddAB proteins (and their orthologs RexAB) replace RecBCD in firmicutes and in most β- and α-proteobacteria. AddAB is almost ubiquitous among these groups, as it is missing only in *Bacillus halodurans, Neisseria meningitidis,* and *Chromobacterium violaceum*—these having RecBCD instead. A recent work analyzed a homolog of AddA in proteobacteria and confirmed its role in the repair of double-strand breaks [49]. Although AddA and AddB closely co-occur in most genomes, the AddB gene of *B. subtilis* has no significant similarity with the ones of proteobacteria (*E* > 0.01 for FASTA hits, <25% identity on a global alignment). Because AddB is slightly more conserved than AddA among firmicutes (see following), one would expect the AddB protein of proteobacteria to have significant similarity with the AddB protein of firmicutes if it shared a common evolutionary history. Hence, the AddB proteins of the two clades may be functional analogs but not othologs. This is consistent with recent data indicating that AddA shares stronger resemblance with RecB than AddB does with RecC, reflecting a more central role for the function of RecB/AddA in the complex (M. El Karoui, personal communication).

**Figure 4:**
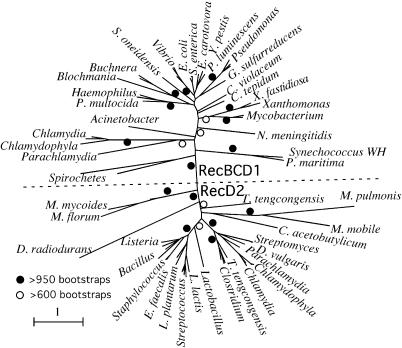
Unrooted Phylogenetic Tree of the RecD Protein The dotted line separates genomes containing RecBCD from the ones containing only RecD. The tree was constructed using Tree-Puzzle, using the JTT+Γ model with eight classes [74]. Bootstraps were made using SEQBOOT and CONSENSE from the PHYLIP package [101]. *C. acetobutylicum, Clostridium acetobutylicum; C. tepidum, Chlorobium tepidum; C. violaceum, Chromobacterium violaceum; D. vulgaris, Desulfovibrio vulgaris; E. carotovora, Erwinia carotovora; E. faecalis, Enterococcus faecalis; L. plantarum, Lactobacillus plantarum; L. lactis, Lactococcus lactis; P. multocida, Pasteurella multocida; M. mobile, Mycoplasma mobile; M. florum, Mesoplasma florum; M. mycoides, Mycoplasma mycoides; M. pulmonis, Mycoplasma pulmonis; P. maritima, Procholorcoccus maritima; S. enterica, Salmonella enterica; S. oneidensis, Shewanella oneidensis; X. fastidiosa, Xylella fastidiosa.*

Genes coding for proteins that participate in complexes tend to systematically co-occur in genomes. This is the case for AddAB, RuvAB, RuvAB/RuvC(RecU), SbcCD, and MutS1L (see following). A major exception to this trend is the frequent presence of a RecD protein when RecBC is absent, in mollicutes, firmicutes, *D. radiodurans,* both *Streptomyces*, and *Des. vulgaris*. The phylogenetic tree of this protein (Figure 4) shows a clear separation between RecD1 (a protein systematically associated with RecBC) and RecD2 (a protein present in genomes lacking RecBC). Within each RecD group, one can identify most of the major phylogenetic groups of bacteria. For example, among actinobacteria, the *Mycobacterium* (with RecBC) and the two *Streptomyces* (without) are on opposite sides of the tree, and a similar contrast is found in δ-proteobacteria, where *Geobacter sulfurreducens* has RecBC and *Des. vulgaris* does not. In some genomes, such as Chlamydiacea, there are multiple copies of RecD, typically one in each side of the tree. The analysis of the protein sequences of the two groups of RecD shows a major difference between them. RecD2 contains an N-terminus extension including a domain identified as RuvA domain 2–like in InterPro that is absent from RecD1. This domain is also present in UvrC and is essential for the 5′ incision in the prokaryotic nucleotide excision repair process [50]. The RecD2 protein of *D. radiodurans,* the only one biochemically studied, is a DNA helicase with a low processivity and a yet-unidentified role [51].

Finally, some bacteria have a functional nonhomologous end-joining mechanism (NHEJ), allowing the repair of dsDNA breaks [52]. Contrary to homologous recombination, NHEJ does not require sequence homology—only complementary ends. The key factors of NHEJ are a Ku protein that binds to the termini of the double-strand breaks and has the bridging activity, and a ligase that ligates the termini. Our results indicate that NHEJ genes are present in few bacteria (Ku is present in 24 genomes out of the 117), with no particular phylogenetic trend, as they are found in firmicutes, actinobacteria, and several groups of proteobacteria (see Figure 2). As indicated previously [53,54], the two genes tend to co-occur contiguously in genomes, probably constituting an operon. In some bacteria, we found many copies of the Ku/ligase genes. For example, *Agrobacterium tumefaciens* contains six copies of the Ku gene and eight copies of the ligase, and *Bradyrhizobium japonicum* contains four copies of the Ku gene and two copies of the ligase. Thus, in these genomes, Ku has probably a very important role. We then tested the patterns of co-occurrence of NHEJ and RecBCD/AddAB to see whether the presence of one could compensate for the absence of the other (as both act to repair double-strand breaks). We found these systems to co-occur independently (*p* = 0.6, χ^2^ test). NHEJ is the major pathway for repairing DNA double-strand breaks in mammalian cells, whereas homologous recombination is so in yeast [55]. Because most bacterial genomes lack NHEJ, homologous recombination also appears to be the major repair pathway acting on such lesions in bacteria.

### The RecFOR Presynaptic Proteins

Whereas the RecB, RecC, and RecD polypeptides form a stable active complex, in the RecFOR pathway, there are interactions between some of the elements but no stable complexes between the three proteins. Interestingly, the RecBCD/AddAB and RecFOR proteins, instead of showing a complementary pattern of co-occurrence, tend to co-occur more frequently than expected (*p* < 0.001, χ^2^ test). This means that if RecBCD/AddAB is present (absent), then RecFOR is more likely to be present (absent), which probably reflects the specificity of these two systems on complementary types of lesions (see Figure 1).

Although RecF historically served as a reference for this pathway, it is absent from 29 genomes and is the least frequent protein in the set (see Figure 2). At the other extreme, RecR is the most frequent, being absent from only ten genomes, followed by RecO, which, counting putative orthologs, is only absent from 19 genomes. In agreement with RecR being present in the two active complexes RecOR and RecFR [18,19], there is no single occurrence of RecO or RecF when RecR is absent.

In *E. coli,* the RecJ exonuclease acts during gap repair to enlarge the ssDNA region for RecFOR binding [56]. RecJ is absent from the species that lack RecA and from the mollicutes and the mycobacteria, which may use an alternative exonuclease. RecQ is absent from 48 genomes, in agreement with the observation that the RecQ helicase is required in *E. coli* for RecFOR-mediated recombination only in a *recBC sbcB sbcCD* mutant [57].

### Recombination without Presynaptic Recombination Proteins?

Our analysis indicates that certain bacterial genomes lack most presynaptic recombination proteins (see Figure 2). One possibility is that these genomes lack homologous recombination altogether. This may be the case for some species lacking nearly all homologous recombination proteins, such as all *Buchnera,* or the OY phytoplasma (Table 1). However, for the genomes containing RecA and resolvases, this is most unlikely. We therefore made an extensive analysis of the literature and selected genomes lacking most presynaptic proteins but for which there is evidence for homologous recombination (Table 2). Such evidence comes from experimental studies of the homologous recombination processes or experimental studies that have used homologous recombination to engineer/inactivate genes, and from multilocus sequence typing data that indicate a population structure driven by frequent recombination. One also typically assumes that natural transformation is used for recombination repair or gene acquisition, which suggests that competent bacteria should have some type of homologous recombination [4,58]. It is surprising that highly recombining genomes, such as *Helicobacter pylori* [59–61] or *Streptomyces coelicolor* [62] lack a large fraction of the presynaptic proteins. One should note that with the exception of both *Streptomyces,* these genomes also lack NHEJ, and many also code for antirecombinants, such as MutS2. This suggests that either presynaptic proteins are dispensable for efficient homologous recombination in some genomes or other, unknown systems, exist in these genomes. The first hypothesis is supported by data indicating that some *E. coli recA* mutations (RecA P67W, RecA441, RecA730, and RecA803) can displace SSB proteins much more efficiently than the wild-type, and thus function in the absence of presynaptic proteins [63]. However, if some genomes lack presynaptic functions because their RecA protein is able to efficiently bind SSB-covered DNA, it is not through one of the studied RecA mutations in *E. coli,* because we did not find any of these mutations in natural genomes. Furthermore, it remains to be understood how organisms lacking presynaptic functions could control RecA activity to avoid its improper fixation to any ssDNA (e.g., on the template of the replicating lagging strand). Yet-unidentified presynaptic systems may exist in these genomes. Recombination presynaptic functions are fulfilled in eukaryotes by proteins that have no homology with *E. coli* proteins, in spite of their capacity to facilitate the binding to DNA of their cognate RecA homolog [64].

**Table 2:**
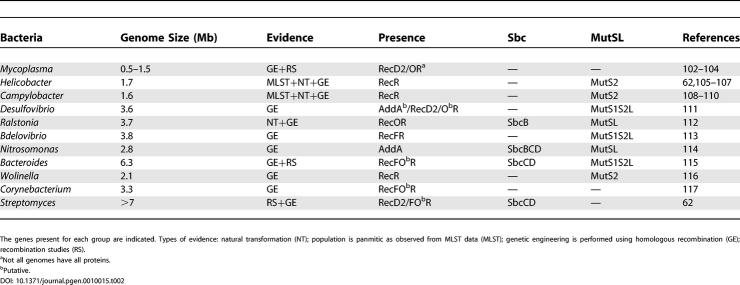
Bacteria for Which Some Evidence Exists of Homologous Recombination, but That Lack Elements of Both RecBCD/AddAB and RecFOR in the Genome

The genes present for each group are indicated. Types of evidence: natural transformation (NT); population is panmitic as observed from MLST data (MLST); genetic engineering is performed using homologous recombination (GE); recombination studies (RS).

^a^Not all genomes have all proteins.

^b^Putative.

### Proteins That Antagonize Homologous Recombination

Another way of increasing the frequency of homologous recombination without making changes in the recombination machinery is to eliminate the function of antirecombinant proteins. We tested whether there are associations between the losses of presynaptic systems and the losses of antirecombinant proteins, such as UvrD, MutS1L, MutS2, and SbcB genes. UvrD is nearly ubiquitous. The presence of MutS1L correlates with the presence of RecBCD/AddAB and RecFOR (RecBCD/AddAB: observed 102, expected 69; RecFOR: observed 91, expected 80; both *p* < 0.005, Pearson's exact test). This suggests that a lower activity of RecA in the absence of presynaptic systems can be compensated for by the loss of the mismatch repair system. Contrary to MutS1, MutS2 is not involved in mismatch repair and suppresses homologous recombination between identical sequences, in addition to homeologous recombination, in *H. pylori* [60]. However, no significant association was found between the presence or absence of MutS2 and that of the presynaptic systems. As the *H. pylori* enzyme is the only MutS2 that has been studied in detail so far, it is possible that the antirecombination property of this MutS2 protein is specific for this species.

SbcB, which in RecBC^−^ backgrounds prevents the repair of double-strand breaks by RecFOR, has a statistically significant pattern of co-occurrence and co-omission with RecBCD/AddAB (observed 63, expected 53, *p* < 0.01, Pearson's exact test), but not with RecFOR (*p* > 0.1, same test). In fact, only one of the bacteria lacking RecBC/AddAB contains SbcB. This indicates that the absence (presence) of RecBCD/AddAB is correlated with the absence (presence) of this antirecombinant gene, which may allow RecFOR to efficiently repair double-strand breaks in RecBCD^−^/AddAB^−^ backgrounds. SbcCD is much more frequent than SbcB and also co-occurs with RecBCD/AddAB (observed 64, expected 52, *p* < 0.01, Pearson's exact test). However, the role of SbcCD in homologous recombination is unclear.

### Colocalization of Genes

Genes involved in a common mechanism tend to be tightly coregulated and, for this reason, clustered in the genome [65]. We have therefore searched for the colocalization of these genes among our set of genomes. With few exceptions, we found that only the recombination genes that are part of stable complexes are systematically clustered. The *addAB* genes colocalize in 20 of 21 co-occurrences among firmicutes, the exception being *Clostridium tetani*. Among proteobacteria these genes are together in 13 of 13 genomes. The three genes for RecBCD were found to colocalize in 28 of their 31 co-occurrences. RuvA and RuvB colocalized in 77 of 111 co-occurrences, with exceptions including all chlamydiacea, all cyanobacteria, all ɛ-proteobacteria, all streptococci, all bacteroides, and most spirochetes, as well as a few phylogenetically dispersed genomes. RuvA, RuvB, and RuvC colocalized in 45 of 78 co-occurrences of the three genes. In firmicutes and mollicutes, RuvC is replaced by RecU, but this gene only colocalizes with RuvAB in two genomes (*Mycoplasma genitalium* and *M. pneumoniae*). Thus, RecU and RuvC are very different in this respect. YqgF was rarely found close to other recombination genes. The two key genes for NHEJ (Ku and the ligase) were found together in 19 of 24 genomes. Naturally, as for the co-occurrence of genes in genomes, the closeness of their co-occurrence is influenced by the phylogenetic distribution of the available genomes. Close occurrence of genes in highly sampled clades, e.g., firmicutes or proteobacteria, will be more preeminent than in clades with few available sequences.

RecA and RecX are close in many genomes and are partly coexpressed in *E. coli* [66]. In some bacteria, the overexpression of RecA is toxic in the absence of RecX, and in vitro, RecX modulates the action of RecA by blocking the extension of the RecA filament [67]. However, although in *E. coli* RecX inhibits the action of RecA [68], in *Neisseria gonorrhoeae* its inactivation leads to a decrease in homologous recombination [66]. Expanding previous observations [69], we found that 35 of the 37 co-occurrences of bona fide orthologs of *recX* colocalize with *recA*. The exceptions are *N. meningitidis* and *Photorhabdus luminescens*. In contrast, very few genes among the more distantly related, putative *recX* orthologs are physically close to *recA* genes. In particular, the putative *recX* of firmicutes are systematically far in the chromosome from *recA*. The proteins coded by these genes are larger and less than 40% similar to the RecX from *E. coli* and from actinobacteria. It is thus uncertain whether they perform the same function. However, RecX also shows large relative variations in length among well-characterized orthologs (e.g., among γ-proteobacteria the *E. coli* protein has 166 residues, whereas in *Yersinia pestis* it has 188, and in *Shewanella oneidensis* it has 123). It has been suggested that the uncoupling between *recA* and the putative *recX* in *N. gonorrhoeae* and *B. subtilis* could be associated with their competence for natural transformation [66]. However, such uncoupling is a characteristic of all firmicutes, not specifically of the competent ones, and it is not found in other competent bacteria such as *Haemophilus influenzae* or *H. pylori* (which lacks RecX).

Although *recF, recR,* and *recO* do not colocalize, both *recF* and *recR* often colocalize with genes coding for replication proteins. Many genomes have an operon close to the replication origin containing four genes: *dnaA* (involved in replication initiation), *dnaN* (β-clamp of the DNA polymerase III), *recF,* and *gyrB* (DNA gyrase) [70]. Among the 86 occurrences of *recF,* it is close to *dnaA* in 54, close to *dnaN* in 58, and close to *gyrB* in 52. The four genes are together in 40 genomes. Finally, the *dnaX* gene, which encodes both the τ and γ subunits of *E. coli* DNA polymerase III, is close to *recR* in *E. coli,* and the genes are partially cotranscribed [71]. Among the 97 genomes containing *dnaX* and *recR,* the genes colocalize in 65. These results indicate that instead of clustering together, recombination genes that are not part of stable complexes are often colocalized with genes involved in replication. The linkage between genes of these two cellular processes is certainly associated with the role of homologous recombination in repairing DNA lesions that block DNA synthesis [72,73].

### Relative Evolutionary Rates of the Proteins

**Figure 5:**
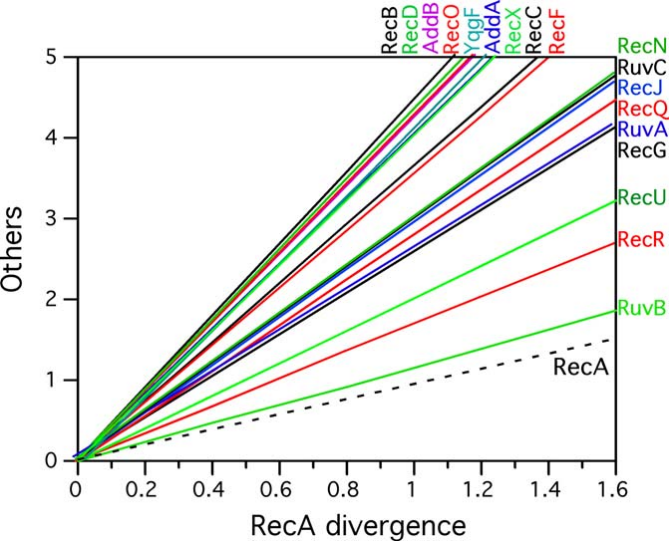
Regression Lines of the Substitution Rates of the Recombination Proteins Plotted against the Substitution Rates of RecA RecA is the slowest and most ubiquitous of these proteins and its substitution rates are the *x*-axis of the plot (dashed lines indicate the RecA identity line). The regression was forced to pass through zero at intercept, and the slopes of the lines indicate the relative rapidity of the protein's evolution relative to that of RecA (varies from 1.3 for RuvB to 4.4 to RecB); the points associated with mollicutes were removed because we found these to evolve proportionally faster in RecA than in the other proteins.

The substitution rate of proteins is the result of the interplay between mutation and functional constraints. Hence, if one discounts horizontal gene transfer, the differences in substitution rates between proteins should reflect their relative tolerance to change (i.e., they should be associated with the fraction of changes that allows maintaining the function). To assess the relative tolerance of each recombination protein to changes, we computed evolutionary distances within the sets of all bona fide orthologs, using Tree-Puzzle [74]. We then used RecA as the reference protein because of its near ubiquity and slow evolutionary rate [42]. The regression analyses of the substitution rates of each protein as a function of the substitution rate of RecA showed one single group in which RecA evolves faster—the mollicutes (data not shown). We have thus not used these points in the regressions. All other proteins were then compared to RecA, and we found a considerable diversity among the different proteins in terms of substitution rates (Figure 5). A more developed version of this method has recently been proposed to find horizontal gene transfer between distant taxa [75]. Using our data, we found very little evidence of such events (data not shown). RuvB has evolved almost as slowly as RecA (16% faster), whereas some proteins have evolved a little faster, such as RecR (+68%) and RecU (+100%). However, most proteins have evolved much faster than RecA. Among these, there is a group of proteins that has evolved between 4.0 and 4.5 times faster than RecA and that includes RecB, RecD, RecX, AddA, AddB, YqgF, and RecO. Because RecD is divided in two groups, these data only include the RecD proteins that are in the group of genomes containing RecBC (i.e., RecD1).

The proteins of the RecFOR pathway have a peculiar evolutionary pattern. In addition to being present with very different frequency, with RecF being more frequently absent than RecR or RecO, they also show remarkably different substitution rates, with high conservation for RecR, lower conservation for RecF, and among the lowest conservation for RecO (Figure 5). This may be the result of the double participation of RecR in interactions with RecO and RecF, which would increase the constraints on its evolution. The crystal structure of the *D. radiodurans* RecR protein reveals the existence of a ring-shaped tetramer, theoretically able to encircle dsDNA [76]. This particular clamp-like structure may also have contributed to the high level of conservation of the protein.

**Figure 6:**
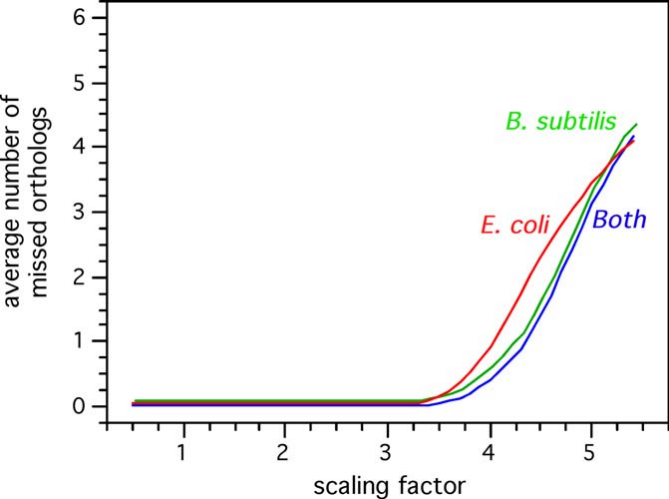
Results of the Simulations of Protein Evolution following the Phylogenetic Tree of RecA Using the JTT+Γ Model with Eight Classes and an α of 0.59 We used Seq-Gen to evolve protein sequences with regularly spaced scaling factors (100 experiments for each scaling factor) and analyzed for each experience which sequences showed less than 40% similarity.

It's interesting to note that among the fastest-evolving proteins, some are nearly ubiquitous (RecD and YqgF), and some are much rarer (RecB and AddAB). This suggests that few proteins have been missed in the analysis as a result of excessive sequence divergence. We made a set of simulations to assess this problem more precisely. We allowed protein sequences to evolve according to the evolutionary model of RecA, but at a different relative rates (see Materials and Methods). This analysis showed that only proteins evolving more than four times faster than RecA are expected to be missed in our similarity searches at this evolutionary distance and using our 40% similarity criterion (Figure 6). Even for proteins evolving 5.5 times faster than RecA, in none of our 100 simulations would we miss more than six orthologs. These orthologs were systematically in the fast-evolving mollicutes clade. Naturally, this is an oversimplification of the evolution of proteins, because proteins evolve in a changing context, and this may change their relative rates of evolution. In addition, these analyses do not take into account that insertions and deletions may be more frequent in some proteins than in others. Yet they indicate that few homologous genes are expected to have been lost in the present analysis as a result of excessive sequence divergence.

### Conclusion

The presynaptic role of RecBCD and RecFOR and the branch migration activity of RuvAB and RecG suggest functional redundancy, whereas, in contrast, the patterns of co-occurrence of these systems agree with the experimental works indicating complementary, and not redundant, roles for these proteins. Interestingly, this work also indicates that the RecFOR pathway may be more conspicuously important among bacteria than RecBCD, as it is significantly more frequent. RecR is the most conserved of the three proteins, and understanding how recombination is promoted in the organisms that encode a RecR homolog but do not have RecF or RecO would help understand the functioning of these recombination mediator proteins. The associations of *recR* and *recF* with genes involved in replication are often conserved, suggesting that the close association between replication and recombination observed in *E. coli* is common to most bacteria.

A central tenet of current genomic studies is the possibility of associating gene content with phenotype variation. Because the abundance of repeats in genomes correlates well with rearrangement rates and with the capacity of generating genetic variation [8,9], and because repeats are cause and consequence of recombination processes, one could expect an association between the repertoire of recombination genes and the number of repeats. We were unable to observe such a correlation. Indeed, except for genomes lacking RecA and resolvases (which are stable, have few repeats, and possibly lack homologous recombination), bacteria known to recombine frequently may either have a complete repertoire of known recombination genes or lack a substantial part of it. A striking example of the latter is provided by *H. pylori* [77], which is highly recombinogenic, although it lacks most presynaptic proteins and has antirecombinants such as UvrD and MutS2. In addition, at the intraspecies level, the differences in the population structure do not correlate with the genome content in recombination proteins. For example, serogroup A of *N. meningitidis* is mostly clonal, contrary to the majority of the others [78]. However, we found that both serotypes A [79] and B [80] have the same almost complete repertoire of homologous recombination proteins. Hence, associations between stability of a genome and the lack of some recombination proteins, as was proposed for *Bifidobacterium longum* [81] and *Corynebacterium* species [38], must be viewed with exceptional care before experimental confirmation.

The reasons for this lack of simple association between genotype and phenotype are probably multiple. Orthologs do not necessarily have the same exact functionalities and are likely to have different levels of activity. For example, presynaptic systems may be less necessary if the affinity of RecA for ssDNA is higher. The frequency of recombination events may also depend on the implication of recombination proteins in different cellular processes. For example, the coupling of recombination and replication may depend on the replication machinery and on the frequency of replication arrest. Specific genetic regulatory systems may also lead to different rates of recombination. For example, the onset of competence may be differently related in various organisms with cell growth and with the level of expression of recombination enzymes. Also, equivalent cellular processes may be associated with different enzymatic systems. For example, in neisserial species and *E. coli,* transformation-associated recombination takes place through the RecBCD pathway, whereas in *B. subtilis,* chromosomal transformation decreases 2.5-fold in a *recO* mutant [82], and in streptococci, AddAB is not involved in chromosome transformation [83], possibly because in competent firmicutes only ssDNA enters the cell. In contrast, in the competent *Helicobacter* and *Campylobacter* species, all these genes but RecR are absent. One could also expect that recombination activity is also constrained by ecological factors. Endosymbionts live in very protected environments, and this, associated with reductive genome evolution, has led to the loss of recombination functions [36,37]. However, apart from this case, we could not find any other obvious association between lifestyle and the presence or absence of recombination proteins, which once again is in agreement with the inherent housekeeping role of homologous recombination.

This housekeeping role of homologous recombination is probably also why we found little evidence of horizontal transfer among these genes. Genes implicated in the generation of genetic variation tend to be frequently horizontally transferred [84,85], but not housekeeping genes involved in managing genetic information [86]. Interestingly, multilocus sequence data also indicate that RecA rarely recombines among strains of the same species [87,88]. This does not mean that horizontal transfer is altogether absent. Such events are the most parsimonious explanation for the existence of some analogous replacements, such as AddAB among proteobacteria or RuvC in *Thermoanaerobacter tengcongensis*. They are also probably responsible for the sporadic occurrence of NHEJ in different phylogenetic groups. In addition, given the frequency of prophage sequences in bacterial genomes [89], and the many phage-encoded recombination systems, recombination genes of known phage origin, which have not been included in this study, may also play a role in the variations of recombination mechanisms.

Our study defines a core of recombination genes coding for proteins nearly ubiquitous in bacterial species. These include the genes that encode RecA (which has a homolog among eukaryotes), RuvAB, RecR, RuvC/RecU, and to a minor extent RecG, RecN, RecJ, and PriA. These genes are present in nearly all bacterial groups and show little horizontal transfer. This justifies the use of such proteins as phylogenetic markers [43]. Their widespread distribution demonstrates their importance in bacteria and justifies the emphasis on their detailed biochemical and functional study.

## Materials and Methods

### Data.

We analyzed the genomes of 117 different bacterial species (see Figure 2), taken from GenBank Genomes (ftp://ftp.ncbi.nih.gov/genomes/Bacteria/). The list of proteins related to homologous recombination was taken from the literature [13,24] and included RecA, RecB, RecC, RecD, RecF, RecG, RecJ, RecN, RecO, RecQ, RecR, RecU, RecX, RuvA, RuvB, RuvC, AddA (RexA), AddB (RexB), and PriA. Their function is summarized in Figure 1. Proteins such as RecE, RecT, and RusA were not analyzed because they were found to be very rare in bacterial genomes and are associated with prophages [13]. In addition, we included the antirecombination proteins SbcB, SbcC, SbcD*,* MutS1, MutS2, MutL, and UvrD; the putative resolvase YqgF [47]; and the Ku and ligase genes responsible for nonhomologous end joining in some bacteria [52].

### Assignment of orthology.

One should note that many recombination genes belong to large protein families, such as helicases [90] or nucleases [47]. Hence, simple sequence similarity is not an indication of orthology. Assignment of orthology followed an automated step and then manual curation. The automatic method was the following. We started from the protein in *E. coli* (except for AddAB, MutS2, Ku, and RecU, where we started from *B. subtilis*) and searched for orthology in all other genomes. Genes were regarded as potential orthologs if they were bidirectional best hits with at least 40% similarity in sequence and their sequences were less than 30% different in length. The alignments were done using an adapted version of the Neddleman-Wusch algorithm (global alignment), in which the nonaligned edges of the largest sequence are not penalized [91], using the matrix BLOSUM60 and typical gap penalties. For comparison, we also made FASTA searches, because they allow for the detection of more local similarities [92]. Then we took the less similar protein hit, respecting the previously cited conditions as a query, and relaunched the analysis on the entire set of genomes with the same parameters. The proteins resulting from the intersection of these lists were temporarily regarded as bona fide orthologs. The other proteins were put together with the ones showing significant FASTA hits (*E* < 10^−5^) on the other genomes, as well as the ones originally annotated as orthologs (but not respecting the above conditions). We then searched for significant motifs in this set of proteins, using the InterPro database (http://www.ebi.ac.uk/interpro/) and visually analyzed and corrected multiple alignments. The proteins showing alignments with more than 40% similarity with bona fide orthologs were kept. When the alignments were within the range of 37%–40% similarity and did not show excessive gaps, and the proteins respected the 30% difference in length criterion or had significant InterPro motifs, the proteins were classed as putative. The bona fide orthologs were then aligned and phylogenetic distances computed as described below. The final list of “bona fide orthologs” took into account not only sequence similarity searches but also the phylogenetic information and colocalization data, as recommended [93].

### Phylogenetic analyses and simulations of protein evolution.

Orthologs were aligned using ClustalW [94] and checked with Seaview [95]. Phylogenetic distances between the orthologous proteins were computed using Tree-Puzzle [74], with the JTT+Γ model with eight classes. For this analysis, and because we wanted to assess evolutionary rates, we removed only the regions with extended gaps from the multiple alignments. Phylogenetic trees were built using the same model with Phyml [96]. We used Seq-Gen [97] to generate 1,000 proteins with 1,000 residues, having the average amino acid composition of the JTT substitution matrix. The sequences were made to evolve along the RecA phylogenetic tree (which is largely congruent with the 16S rDNA tree [42]), using scaling factors in the range 0.5 to 6 (the fastest protein was found to evolve at less than 4.5 times the rate of RecA), and with the evolutionary model used to build the RecA tree. Each time, we used the evolved sequences to make global alignments and compute the similarity. For each experience, we counted how many genes had more than 40% and more than 37% similarity with the *E. coli* gene. This allowed the assessment of the number of orthologs that may be missed by the automatic similarity search part of the methods as a result of excessive sequence divergence.

### Colocalization analysis.

Two genes were considered to closely co-occur if they were fewer than five genes away in a genome. A third gene is in close co-occurrence with the latter two if it is less than five genes away from at least one of the two genes. One should note that the average operon in *E. coli* and *B. subtilis* has fewer than five genes [98]. We started by analyzing the co-occurrence of the orthologs of the *E. coli* recombination genes. Then we did the same with the orthologs of *B. subtilis* genes that have no orthologue in *E. coli*. Finally, we analyzed particular cases described in the literature: the occurrence of *recF* in the *dnaA* region [70] and the co-occurrence of *recR* with *dnaX* [71], and *recX* with *recA* [69].

## Abbreviations

dsDNA: double-stranded DNA
NHEJ: nonhomologous end-joining mechanism
OY: Onion Yellows
ssDNA: single-stranded DNA

## References

[pgen-0010015-b01] Kuzminov A (1999). Recombinational repair of DNA damage in *Escherichia coli* and bacteriophage lambda. Microbiol Mol Biol Rev.

[pgen-0010015-b02] Michel B, Grompone G, Florès MJ, Bidnenko V (2004). Multiple pathways process stalled replication forks. Proc Natl Acad Sci U S A.

[pgen-0010015-b03] Smith GR (1991). Conjugational recombination in *E. coli:* Myths and mechanisms. Cell.

[pgen-0010015-b04] Lorenz MG, Wackernagel W (1994). Bacterial gene transfer by natural genetic transformation in the environment. Microbiol Rev.

[pgen-0010015-b05] Feil EJ (2004). Small change: Keeping pace with microevolution. Nat Rev Microbiol.

[pgen-0010015-b06] Otto SP, Michalakis Y (1998). The evolution of recombination in changing environments. Trends Ecol Evol.

[pgen-0010015-b07] Hughes D, Charlebois RL (1999). Impact of homologous recombination on genome organization and stability. Organization of the prokaryotic genome.

[pgen-0010015-b08] Rocha EPC (2004). Order and disorder in bacterial genomes. Curr Opin Microbiol.

[pgen-0010015-b09] Rocha EPC (2003). DNA repeats lead to the accelerated loss of gene order in bacteria. Trends Genet.

[pgen-0010015-b10] Finlay BB, Falkow S (1997). Common themes in microbial pathogenicity revisited. Microbiol Mol Biol Rev.

[pgen-0010015-b11] Mehr IJ, Seifert HS (1998). Differential roles of homologous recombination pathways in *Neisseria gonorrhoeae* pilin antigenic variation, DNA transformation, and DNA repair. Mol Microbiol.

[pgen-0010015-b12] Rocha EPC, Blanchard A (2002). Genomic repeats, genome plasticity and the dynamics of *Mycoplasma* evolution. Nucleic Acids Res.

[pgen-0010015-b13] Kowalczykowski SC, Dixon DA, Eggleston AK, Lauder SD, Rehrauer WM (1994). Biochemistry of homologous recombination in *Escherichia coli*. Microbiol Rev.

[pgen-0010015-b14] Cox MM (2002). Historical overview: Searching for replication help in all the rec places. Proc Natl Acad Sci U S A.

[pgen-0010015-b15] Konforti BB, Davis RW (1990). The preference for a 3′ homologous end is intrinsic to RecA-promoted strand exchange. J Biol Chem.

[pgen-0010015-b16] Singleton MR, Dillingham MS, Gaudier M, Kowalczykowski SC, Wigley DB (2004). Crystal structure of RecBCD enzyme reveals a machine for processing DNA breaks. Nature.

[pgen-0010015-b17] Kowalczykowski SC (2000). Initiation of genetic recombination and recombination-dependent replication. Trends Biochem Sci.

[pgen-0010015-b18] Webb BL, Cox MM, Inman RB (1997). Recombinational DNA repair: The RecF and RecR proteins limit the extension of RecA filaments beyond single-strand DNA gaps. Cell.

[pgen-0010015-b19] Morimatsu K, Kowalczykowski SC (2003). RecFOR proteins load RecA protein onto gapped DNA to accelerate DNA strand exchange: A universal step of recombinational repair. Mol Cell.

[pgen-0010015-b20] Lusetti SL, Cox MM (2002). The bacterial RecA protein and the recombinational DNA repair of stalled replication forks. Annu Rev Biochem.

[pgen-0010015-b21] Goodman MF, Tippin B (2000). Sloppier copier DNA polymerases involved in genome repair. Curr Opin Genet Dev.

[pgen-0010015-b22] Marians KJ (2000). PriA-directed replication fork restart in *Escherichia coli*. Trends Biochem Sci.

[pgen-0010015-b23] Polard P, Marsin S, McGovern S, Velten M, Wigley DB (2002). Restart of DNA replication in Gram-positive bacteria: Functional characterisation of the *Bacillus subtilis* PriA initiator. Nucleic Acids Res.

[pgen-0010015-b24] Dubnau D, Lovett CM, Sonenshein AL, Hoch JA, Losick R (2002). Transformation and recombination. Bacillus subtilis and its closest relatives.

[pgen-0010015-b25] el Karoui M, Ehrlich D, Gruss A (1998). Identification of the lactococcal exonuclease/recombinase and its modulation by the putative Chi sequence. Proc Natl Acad Sci U S A.

[pgen-0010015-b26] El Karoui M, Biaudet V, Schbath S, Gruss A (1999). Characteristics of Chi distribution on different bacterial genomes. Res Microbiol.

[pgen-0010015-b27] Ayora S, Carrasco B, Doncel E, Lurz R, Alonso JC (2004). *Bacillus subtilis* RecU protein cleaves Holliday junctions and anneals single-stranded DNA. Proc Natl Acad Sci U S A.

[pgen-0010015-b28] Feinstein SI, Low KB (1986). Hyper-recombining recipient strains in bacterial conjugation. Genetics.

[pgen-0010015-b29] Vulic M, Dionisio F, Taddei F, Radman M (1997). Molecular keys to speciation: DNA polymorphism and the control of genetic exchange in enterobacteria. Proc Natl Acad Sci U S A.

[pgen-0010015-b30] Morel P, Hejna JA, Ehrlich SD, Cassuto E (1993). Antipairing and strand transferase activities of *E. coli* helicase II (UvrD). Nucleic Acids Res.

[pgen-0010015-b31] Veaute X, Delmas S, Selva M, Jeusset J, Le Cam E (2005). UvrD helicase, unlike Rep helicase, dismantles RecA nucleoprotein filaments in *Escherichia coli*. EMBO J.

[pgen-0010015-b32] Mendonca VM, Matson SW (1995). Genetic analysis of delta helD and delta uvrD mutations in combination with other genes in the RecF recombination pathway in *Escherichia coli:* Suppression of a ruvB mutation by a uvrD deletion. Genetics.

[pgen-0010015-b33] Gibson FP, Leach DR, Lloyd RG (1992). Identification of sbcD mutations as cosuppressors of recBC that allow propagation of DNA palindromes in *Escherichia coli* K-12. J Bacteriol.

[pgen-0010015-b34] Eisen JA, Hanawalt PC (1999). A phylogenomic study of DNA repair genes, proteins, and processes. Mutat Res.

[pgen-0010015-b35] Fraser CM, Norris SJ, Weinstock GM, White O, Sutton GG (1998). Complete genome sequence of *Treponema pallidum* the syphilis spirochete. Science.

[pgen-0010015-b36] Silva FJ, Latorre A, Moya A (2003). Why are the genomes of endosymbiotic bacteria so stable?. Trends Genet.

[pgen-0010015-b37] Dale C, Wang B, Moran N, Ochman H (2003). Loss of DNA recombinational repair enzymes in the initial stages of genome degeneration. Mol Biol Evol.

[pgen-0010015-b38] Nakamura Y, Nishio Y, Ikeo K, Gojobori T (2003). The genome stability in *Corynebacterium* species due to lack of the recombinational repair system. Gene.

[pgen-0010015-b39] Sicheritz-Ponten T, Andersson SG (2001). A phylogenomic approach to microbial evolution. Nucleic Acids Res.

[pgen-0010015-b40] Ingleston SM, Dickman MJ, Grasby JA, Hornby DP, Sharples GJ (2002). Holliday junction binding and processing by the RuvA protein of *Mycoplasma pneumoniae*. Eur J Biochem.

[pgen-0010015-b41] Della M, Palmbos PL, Tseng HM, Tonkin LM, Daley JM (2004). Mycobacterial Ku and ligase proteins constitute a two-component NHEJ repair machine. Science.

[pgen-0010015-b42] Eisen JA (1995). The RecA protein as a model molecule for molecular systematic studies of bacteria: Comparison of trees of RecAs and 16S rRNAs from the same species. J Mol Evol.

[pgen-0010015-b43] Santos SR, Ochman H (2004). Identification and phylogenetic sorting of bacterial lineages with universally conserved genes and proteins. Environ Microbiol.

[pgen-0010015-b44] Shigenobu S, Watanabe H, Hattori M, Sakaki Y, Ishikawa H (2000). Genome sequence of the endocellular bacterial symbiont of aphids *Buchnera* sp. APS. Nature.

[pgen-0010015-b45] Tamas I, Klasson L, Canback B, Naslund AK, Eriksson AS (2002). 50 million years of genomic stasis in endosymbiotic bacteria. Science.

[pgen-0010015-b46] Oshima K, Kakizawa S, Nishigawa H, Jung HY, Wei W (2004). Reductive evolution suggested from the complete genome sequence of a plant-pathogenic phytoplasma. Nat Genet.

[pgen-0010015-b47] Aravind L, Makarova KS, Koonin EV (2000). Survey and summary: Holliday junction resolvases and related nucleases: Identification of new families, phyletic distribution, and evolutionary trajectories. Nucleic Acids Res.

[pgen-0010015-b48] Sharples GJ, Ingleston SM, Lloyd RG (1999). Holliday junction processing in bacteria: Insights from the evolutionary conservation of RuvABC, RecG, and RusA. J Bacteriol.

[pgen-0010015-b49] Zuniga-Castillo J, Romero D, Martinez-Salazar JM (2004). The recombination genes addAB are not restricted to gram-positive bacteria: Genetic analysis of the recombination initiation enzymes RecF and AddAB in *Rhizobium etli*. J Bacteriol.

[pgen-0010015-b50] Singh S, Folkers GE, Bonvin AM, Boelens R, Wechselberger R (2002). Solution structure and DNA-binding properties of the C-terminal domain of UvrC from *E. coli*. EMBO J.

[pgen-0010015-b51] Wang J, Julin DA (2004). DNA helicase activity of the RecD protein from *Deinococcus radiodurans*. J Biol Chem.

[pgen-0010015-b52] Weller GR, Kysela B, Roy R, Tonkin LM, Scanlan E (2002). Identification of a DNA nonhomologous end-joining complex in bacteria. Science.

[pgen-0010015-b53] Aravind L, Koonin EV (2001). Prokaryotic homologs of the eukaryotic DNA-end-binding protein Ku, novel domains in the Ku protein and prediction of a prokaryotic double-strand break repair system. Genome Res.

[pgen-0010015-b54] Doherty AJ, Jackson SP, Weller GR (2001). Identification of bacterial homologues of the Ku DNA repair proteins. FEBS Lett.

[pgen-0010015-b55] Krejci L, Chen L, Van Komen S, Sung P, Tomkinson A (2003). Mending the break: Two DNA double-strand break repair machines in eukaryotes. Prog Nucleic Acid Res Mol Biol.

[pgen-0010015-b56] Lovett ST, Clark AJ (1984). Genetic analysis of the recJ gene of *Escherichia coli* K-12. J Bacteriol.

[pgen-0010015-b57] Mendonca VM, Klepin HD, Matson SW (1995). DNA helicases in recombination and repair: Construction of a delta uvrD delta helD delta recQ mutant deficient in recombination and repair. J Bacteriol.

[pgen-0010015-b58] Wojciechowski MF, Hoelzer MA, Michod RE (1989). DNA repair and the evolution of transformation in *Bacillus subtilis*. II. Role of inducible repair. Genetics.

[pgen-0010015-b59] Falush D, Kraft C, Taylor NS, Correa P, Fox JG (2001). Recombination and mutation during long-term gastric colonization by *Helicobacter pylori:* Estimates of clock rates, recombination size, and minimal age. Proc Natl Acad Sci U S A.

[pgen-0010015-b60] Pinto AV, Mathieu A, Marsin S, Veaute X, Ielpi L (2005). Suppression of homologous and homeologous recombination by the bacterial MutS2 protein. Mol Cell.

[pgen-0010015-b61] Aras RA, Kang J, Tschumi AI, Harasaki Y, Blaser MJ (2003). Extensive repetitive DNA facilitates prokaryotic genome plasticity. Proc Natl Acad Sci U S A.

[pgen-0010015-b62] Chen CW, Huang CH, Lee HH, Tsai HH, Kirby R (2002). Once the circle has been broken: Dynamics and evolution of *Streptomyces* chromosomes. Trends Genet.

[pgen-0010015-b63] Kowalczykowski SC (1991). Biochemical and biological function of *Escherichia coli* RecA protein: Behavior of mutant RecA proteins. Biochimie.

[pgen-0010015-b64] Aylon Y, Kupiec M (2004). New insights into the mechanism of homologous recombination in yeast. Mutat Res.

[pgen-0010015-b65] Hershberg R, Yeger-Lotem E, Margalit H (2005). Chromosomal organization is shaped by the transcription regulatory network. Trends Genet.

[pgen-0010015-b66] Stohl EA, Seifert HS (2001). The *recX* gene potentiates homologous recombination in *Neisseria gonorrhoeae*. Mol Microbiol.

[pgen-0010015-b67] Drees JC, Lusetti SL, Chitteni-Pattu S, Inman RB, Cox MM (2004). A RecA filament capping mechanism for RecX protein. Mol Cell.

[pgen-0010015-b68] Lusetti SL, Drees JC, Stohl EA, Seifert HS, Cox MM (2004). The DinI and RecX proteins are competing modulators of RecA function. J Biol Chem.

[pgen-0010015-b69] De Mot R, Schoofs G, Vanderleyden J (1994). A putative regulatory gene downstream of recA is conserved in gram-negative and gram-positive bacteria. Nucleic Acids Res.

[pgen-0010015-b70] Yoshikawa H, Ogasawara N (1991). Structure and function of DnaA and the DnaA-box in eubacteria: Evolutionary relationships of bacterial replication origins. Mol Microbiol.

[pgen-0010015-b71] Chen KS, Saxena P, Walker JR (1993). Expression of the *Escherichia coli dnaX* gene. J Bacteriol.

[pgen-0010015-b72] Courcelle J, Carswell-Crumpton C, Hanawalt PC (1997). *recF* and *recR* are required for the resumption of replication at DNA replication forks in *Escherichia coli*. Proc Natl Acad Sci U S A.

[pgen-0010015-b73] Chow KH, Courcelle J (2004). RecO acts with RecF and RecR to protect and maintain replication forks blocked by UV-induced DNA damage in *Escherichia coli*. J Biol Chem.

[pgen-0010015-b74] Schmidt HA, Strimmer K, Vingron M, von Haeseler A (2002). TREE-PUZZLE: Maximum likelihood phylogenetic analysis using quartets and parallel computing. Bioinformatics.

[pgen-0010015-b75] Novichkov PS, Omelchenko MV, Gelfand MS, Mironov AA, Wolf YI (2004). Genome-wide molecular clock and horizontal gene transfer in bacterial evolution. J Bacteriol.

[pgen-0010015-b76] Lee BI, Kim KH, Park SJ, Eom SH, Song HK (2004). Ring-shaped architecture of RecR: Implications for its role in homologous recombinational DNA repair. EMBO J.

[pgen-0010015-b77] Tomb JF, White O, Kerlavage AR, Clayton RA, Sutton GG (1997). The complete genome sequence of the gastric pathogen *Helicobacter pylori*. Nature.

[pgen-0010015-b78] Bart A, Barnabe C, Achtman M, Dankert J, van der Ende A (2001). The population structure of *Neisseria meningitidis* serogroup A fits the predictions for clonality. Infect Genet Evol.

[pgen-0010015-b79] Parkhill J, Achtman M, James KD, Bentley SD, Churcher C (2000). Complete DNA sequence of a serogroup A strain of *Neisseria meningitidis Z2491*. Nature.

[pgen-0010015-b80] Tettelin H, Saunders NJ, Heidelberg J, Jeffries AC, Nelson KE (2000). Complete genome sequence of *Neisseria meningitidis* serogroup B strain *MC58*. Science.

[pgen-0010015-b81] Schell MA, Karmirantzou M, Snel B, Vilanova D, Berger B (2002). The genome sequence of *Bifidobacterium longum* reflects its adaptation to the human gastrointestinal tract. Proc Natl Acad Sci U S A.

[pgen-0010015-b82] Fernandez S, Kobayashi Y, Ogasawara N, Alonso JC (1999). Analysis of the *Bacillus subtilis* recO gene: RecO forms part of the RecFLOR function. Mol Gen Genet.

[pgen-0010015-b83] Halpern D, Gruss A, Claverys JP, El-Karoui M (2004). rexAB mutants in *Streptococcus pneumoniae*. Microbiology.

[pgen-0010015-b84] Kobayashi I (2001). Behavior of restriction-modification systems as selfish mobile elements and their impact on genome evolution. Nucleic Acids Res.

[pgen-0010015-b85] Denamur E, Lecointre G, Darlu P, Tenaillon O, Acquaviva C (2000). Evolutionary implications of the frequent horizontal transfer of mismatch repair genes. Cell.

[pgen-0010015-b86] Rivera MC, Rain R, Moore JE, Lake JA (1998). Genomic evidence for two functionally distinct gene classes. Proc Natl Acad Sci U S A.

[pgen-0010015-b87] Feil E, Zhou J, Maynard Smith J, Spratt BG (1996). A comparison of the nucleotide sequences of the *adk* and *recA* genes of pathogenic and commensal *Neisseria* species: Evidence for extensive interspecies recombination within *adk*. J Mol Evol.

[pgen-0010015-b88] Tu ZC, Dewhirst FE, Blaser MJ (2001). Evidence that the *Campylobacter* fetus sap locus is an ancient genomic constituent with origins before mammals and reptiles diverged. Infect Immun.

[pgen-0010015-b89] Casjens S (2003). Prophages and bacterial genomics: What have we learned so far?. Mol Microbiol.

[pgen-0010015-b90] Tuteja N, Tuteja R (2004). Unraveling DNA helicases. Motif, structure, mechanism, and function. Eur J Biochem.

[pgen-0010015-b91] Erickson BW, Sellers PH, Sankoff D, Kruskal JB (1983). Recognition of patterns in genetic sequences. Time warps, string edits, and macromolecules: The theory and practice of sequence comparison.

[pgen-0010015-b92] Pearson WR (1990). Rapid and sensitive sequence comparison with FASTP and FASTA. Methods Enzymol.

[pgen-0010015-b93] Eisen JA (1998). Phylogenomics: Improving functional predictions for uncharacterized genes by evolutionary analysis. Genome Res.

[pgen-0010015-b94] Thomson JD, Higgins DG, Gibson TJ (1994). Clustal W: Improving the sensitivity of progressive multiple sequence alignment through sequence weighting, positions-specific gap penalties and weight matrix choice. Nucleic Acids Res.

[pgen-0010015-b95] Galtier N, Gouy M, Gautier C (1996). SEAVIEW and PHYLO_WIN: Two graphic tools for sequence alignment and molecular phylogeny. Comput Appl Biosci.

[pgen-0010015-b96] Guindon S, Gascuel O (2003). A simple, fast, and accurate algorithm to estimate large phylogenies by maximum likelihood. Syst Biol.

[pgen-0010015-b97] Rambaut A, Grassly NC (1997). Seq-Gen: An application for the Monte Carlo simulation of DNA sequence evolution along phylogenetic trees. Comput Appl Biosci.

[pgen-0010015-b98] Zheng Y, Szustakowski JD, Fortnow L, Roberts RJ, Kasif S (2002). Computational identification of operons in microbial genomes. Genome Res.

[pgen-0010015-b99] Brochier C, Bapteste E, Moreira D, Philippe H (2002). Eubacterial phylogeny based on translational apparatus proteins. Trends Genet.

[pgen-0010015-b100] Glockner FO, Kube M, Bauer M, Teeling H, Lombardot T (2003). Complete genome sequence of the marine planctomycete *Pirellula* sp. strain 1. Proc Natl Acad Sci U S A.

[pgen-0010015-b101] Felsenstein J (1993). PHYLIP. Phylogeny Inference Package, version 3.6a [computer program].

[pgen-0010015-b102] Dybvig K, Woodard A (1992). Construction of *recA* mutants of *Acholeplasma laidlawii* by insertional inactivation with a homologous DNA fragment. Plasmid.

[pgen-0010015-b103] Sogaard IZ, Boesen T, Mygind T, Melkova R, Birkelund S (2002). Recombination in *Mycoplasma hominis*. Infect Genet Evol.

[pgen-0010015-b104] Cordova CM, Lartigue C, Sirand-Pugnet P, Renaudin J, Cunha RA (2002). Identification of the origin of replication of the *Mycoplasma pulmonis* chromosome and its use in oriC replicative plasmids. J Bacteriol.

[pgen-0010015-b105] Kuipers EJ, Israel DA, Kusters JG, Blaser MJ (1998). Evidence for a conjugation-like mechanism of DNA transfer in *Helicobacter pylori*. J Bacteriol.

[pgen-0010015-b106] Suerbaum S, Maynard-Smith J, Bapumia K, Morelli G, Smith NH (1998). Free recombination within *Helicobacter pylori*. Proc Natl Acad Sci U S A.

[pgen-0010015-b107] de Jonge R, Bakker D, van Vliet AH, Kuipers EJ, Vandenbroucke-Grauls CM (2003). Direct random insertion mutagenesis of *Helicobacter pylori*. J Microbiol Methods.

[pgen-0010015-b108] Wilson DL, Bell JA, Young VB, Wilder SR, Mansfield LS (2003). Variation of the natural transformation frequency of *Campylobacter jejuni* in liquid shake culture. Microbiology.

[pgen-0010015-b109] Schouls LM, Reulen S, Duim B, Wagenaar JA, Willems RJ (2003). Comparative genotyping of *Campylobacter jejuni* by amplified fragment length polymorphism, multilocus sequence typing, and short repeat sequencing: Strain diversity, host range, and recombination. J Clin Microbiol.

[pgen-0010015-b110] Boer P, Wagenaar JA, Achterberg RP, Putten JP, Schouls LM (2002). Generation of *Campylobacter jejuni* genetic diversity in vivo. Mol Microbiol.

[pgen-0010015-b111] Fu R, Voordouw G (1997). Targeted gene-replacement mutagenesis of dcrA, encoding an oxygen sensor of the sulfate-reducing bacterium *Desulfovibrio vulgaris* Hildenborough. Microbiology.

[pgen-0010015-b112] Bertolla F, Van Gijsegem F, Nesme X, Simonet P (1997). Conditions for natural transformation of *Ralstonia solanacearum*. Appl Environ Microbiol.

[pgen-0010015-b113] Cotter TW, Thomashow MF (1992). A conjugation procedure for *Bdellovibrio bacteriovorus* and its use to identify DNA sequences that enhance the plaque-forming ability of a spontaneous host-independent mutant. J Bacteriol.

[pgen-0010015-b114] Hommes NG, Sayavedra-Soto LA, Arp DJ (1996). Mutagenesis of hydroxylamine oxidoreductase in *Nitrosomonas europaea* by transformation and recombination. J Bacteriol.

[pgen-0010015-b115] Cooper AJ, Kalinowski AP, Shoemaker NB, Salyers AA (1997). Construction and characterization of a *Bacteroides thetaiotaomicron recA* mutant: Transfer of *Bacteroides* integrated conjugative elements is RecA independent. J Bacteriol.

[pgen-0010015-b116] Krafft T, Gross R, Kroger A (1995). The function of *Wolinella succinogenes psr* genes in electron transport with polysulphide as the terminal electron acceptor. Eur J Biochem.

[pgen-0010015-b117] Park SY, Kim HK, Yoo SK, Oh TK, Lee JK (2000). Characterization of *glk,* a gene coding for glucose kinase of *Corynebacterium glutamicum*. FEMS Microbiol Lett.

